# Have we forgotten about heller’s? a case report of childhood disintegrative disorder

**DOI:** 10.1192/j.eurpsy.2021.1674

**Published:** 2021-08-13

**Authors:** K. Devan, S. Austin, S. Chakrabarti

**Affiliations:** 1 Liaison, KINGS COLLEGE, LONDON, United Kingdom; 2 Liaison, King’s College Hospital, LONDON, United Kingdom; 3 Liaison, KINGS COLLEGE HOSPTIAL, LONDON, United Kingdom

**Keywords:** Development, neurology, CAMHS, psychiatry

## Abstract

**Introduction:**

We report on the case of a 15 year old young person with a known diagnosis of autism presenting with a rapid and acute regression in functional abilities, decline in expressive speech and bizarre posturing. The symptoms first started during lockdown (April 2020) with anxiety related to school work followed by urinary incontinence, insomnia, muttering to self and incongruent smiling. Initial medical investigations including MRI, lumbar puncture and 24hour EEG were inconclusive, so she was referred to Paediatric Liaison for assessment.

**Objectives:**

We demonstrate the value of a child psychiatry liaison service being involved with young people in an acute medical hospital

**Methods:**

This young person had a thorough psychiatric assessment.

**Results:**

Through daily psychiatric assessment and reviews with the young person, her parent, social care, wider community team, school and Paediatric Inpatient ward in order to expand on the understanding of the young person and develop a case formulation. She was started on oral Olanzapine 2.5mg which was gradually increased to 10mg OD with minimal improvement.

**Conclusions:**

Childhood Disintegrative Disorder (CDD or Heller’s Syndrome) is a rare pervasive disorder presenting as a loss of previously acquired skills after at least two years of normal development. Despite no longer being included in DSM-V, it is important for Psychiatrists to have a working knowledge of CDD and consider other differentials when assessing young people.

**Disclosure:**

No significant relationships.

